# Dissociation in skin picking disorder and trichotillomania

**DOI:** 10.3389/fpsyt.2025.1490785

**Published:** 2025-04-03

**Authors:** Zharia C. Crisp, Jon E. Grant

**Affiliations:** ^1^ Pritzker School of Medicine, University of Chicago, Chicago, IL, United States; ^2^ Department of Psychiatry & Behavioral Neuroscience, University of Chicago, Chicago, IL, United States

**Keywords:** trichotillomania, skin picking disorder, transdiagnostic, dissociation, hair pulling

## Abstract

**Introduction:**

Dissociation involves a lapse in normal perception of reality or awareness; it has Q6 been associated with multiple psychiatric disorders and has been suggested as a contributing factor in trichotillomania and skin picking disorder. This study aimed to explore the relationship between dissociation and trichotillomania and/or skin picking disorder.

**Methods:**

Three hundred and seventy adults with trichotillomania, skin picking disorder, or both (aged 18-65 years) were enrolled as part of an online survey. Participants completed a questionnaire on demographics, clinical characteristics, comorbidities, medications, and suicidality. Participants completed the Generic Body-Focused Repetitive Behaviors (BFRB) Scale-8 (GBS-8) and the Dissociative Experience Scale (DES). Regression analysis was used to examine the relationship between DES scores and GBS-8 scores, as well as clinical characteristics.

**Results:**

DES scores significantly predicted GBS-8 impairment scores, suicidal ideation, non-suicidal self-injury, and suicide attempts.

**Discussion:**

Dissociation may be either a driving force for many with hair pulling or skin picking, or pulling and picking may create a dissociative trance-like state. These findings suggest that dissociation is associated with greater impairment and worse clinical outcomes. Further research may elucidate whether there is benefit in treating dissociation in these patients.

## 1 Introduction

Dissociation is a fairly common phenomenon in which people feel disconnected from themselves, the world, time, or reality and is due to a failure to integrate perception, memory, cognition and emotions that leads to disruption in normal perception of reality (1). Dissociation may lead to periods where people lack conscious awareness and experience a loss of time, change in location, or engage in behaviors that they do not remember. This phenomenon has been reported across many psychiatric disorders (2), and yet the dissociative experience is poorly understood.

Research has explored dissociation and its relationship with obsessive-compulsive spectrum disorders. Studies have found a relationship between the severity of obsessive- compulsive disorder (OCD) symptoms and frequency of dissociative experiences (1, 3–5) as well as a relationship between time spent gazing in the mirror and resultant dissociation in cases of anorexia nervosa and body dysmorphic disorder (6, 7). Trichotillomania and skin picking disorder have been categorized as obsessive-compulsive related disorders due to several shared features with OCD (8–10), which make them compelling conditions to study in relation to dissociation. Lochner and colleagues 11 found that scores of the Dissociative Experiences Scale (DES) were significantly higher in trichotillomania patients than in skin picking patients. Ozten and colleagues 12 examined dissociation in trichotillomania, skin picking disorder, and healthy controls and found that the three groups did not statistically differ in terms of their scores on the DES.

Given this limited research, as well as a lack of understanding as to whether dissociation relates to symptom severity and clinical characteristics in trichotillomania or skin picking disorder, the purpose of this investigation was to clarify a possible link between dissociation scores and trichotillomania and skin picking disorder. We hypothesized that greater levels of dissociation would be related to worse clinical symptoms of hair pulling and skin picking.

## 2 Methods

### 2.1 Participants

A total of 424 participants were recruited for an online survey using media advertisements and online websites. Inclusion criteria included being an adult aged 18-65 years with a DSM-5 diagnosis of trichotillomania or skin picking disorder. Participants were excluded if they were unable to consent or unable to complete the study due to lack of English fluency. This study and consent statement were approved by the Institutional Review Board of the University of Chicago.

Participants were first required to view the Institutional Review Board (IRB)-approved online informed consent page, at which point an individual could choose to participate in the survey or opt out. The survey asserted that all information was confidential. The survey was open from 4/20/23 to 5/11/23. Participants were compensated for their participation by being included in a raffle where 15 individuals were randomly chosen to receive a $100 gift certificate. Participants were assured that their contact details for the prize draw would be stored separately from their survey responses in order to ensure that their responses were kept completely confidential. Only those individuals completing all measures were reported in the analyses.

Quality checks were performed through rule logic used throughout the survey, which automatically vetted responses for inclusion/exclusion criteria and checked for discrepancies. Research Electronic Data Capture (REDCap) automatically kicked out users who had already completed the survey on a particular device. It also captured the time taken to complete the survey, and people who completed it in <10 minutes were flagged. The data comparison module on REDCap was used after data collection to assess for duplicate/very similar responses. Each response was also reviewed individually to check for inconsistency or very bizarre responses.

### 2.2 Assessments

Participants completed a demographic questionnaire which provided information on their age, sex, gender identity, highest level of education, sexual orientation, race, ethnicity, and annual household income. They were then asked to self-report whether they had a diagnosis of trichotillomania, skin picking disorder, or both, which was verified by their completion of the Minnesota Impulsive Disorders Interview version 2.0 (MIDI 2.0) (13). They also completed a self-report survey assessing history of comorbidities, substance use, current medications, suicidal ideation, and non-suicidal self-injury.

Participants completed the Generic BFRB Scale-8 (GBS-8), an eight-question validated scale which assesses the severity of both trichotillomania and skin picking disorder (α = 0.804) (14). The GBS-8 includes a severity subscale (items 1-4) and an impairment subscale (items 5-8) (α = 0.757 and α = 0.753, respectively).

Lastly, participants completed the DES, a 28-question scale used to measure frequency of various dissociative experiences (α = 0.966) (15). Scores on the DES range from 0 to 100; scores that are over 30 indicate high dissociation, while scores below 30 indicate low dissociation. The DES also consists of three subscales: amnesia, depersonalization/derealization, and absorption. Amnesia factor measures memory loss (i.e., not knowing how you arrived somewhere, finding new items that you don’t recall buying) (α = 0.940). Depersonalization/Derealization factor measures detachment from oneself or a feeling of unreality (i.e., feeling that you’re standing next to yourself, not recognizing yourself in a mirror) (α = 0.913). Absorption factor measures preoccupation with something that is so intense it distracts you from what you’re doing (i.e., memories are so vivid that they feel relived, being so absorbed by a movie that you’re unaware of other things happening) (α = 0.862) (15).

### 2.3 Data analysis

Data analysis was performed using Statistical Package for the Social Sciences (SPSS 29.0). Demographic data and descriptive statistics of the DES were calculated including sample mean and standard deviation (SD). Frequency distribution was also calculated for the DES total score with a standard cutoff score of 30 indicating high dissociation. The relationship between DES score and GBS-8 score was analyzed using linear regression, and the relationship between DES score and various clinical outcomes was analyzed using either linear or binary logistic regression. The significance level for each analysis was set at p < 0.05. One-way ANOVA was used for comparison between groups, and Pearson’s correlation was used to assess the relationship between variables. The Wald Test was used to determine the significance of variables in logistic regression.

## 3 Results

### 3.1 Demographics and descriptive statistics

Data were collected for 424 participants, but 370 participants were included in the final analysis based on exclusion criteria. The average age of participants was 29.2 ± 7.7 years. Other sociodemographic characteristics are presented in **Table 1**.

**Table 1:** Sociodemographic characteristics of participants.

Sample Characteristics	N	%
Sex		
Female	290	78.4
Male	77	20.8
Intersex	3	.8
Gender Identity		
Woman	267	72.2
Man	83	22.4
Nonbinary	13	3.5
Other	2	.5
Not reported	5	1.4
Education		
Less than high school	10	2.7
High school	31	8.4
Some college	79	21.4
Trade school	24	6.5
University degree	140	37.8
Postgraduate degree or more	86	23.2
Sexual Orientation		
Heterosexual	245	66.2
Homosexual	24	6.5
Bisexual	73	19.7
Questioning	5	1.4
Other	20	5.4
Not reported	3	.8
Race		
White	317	85.7
Black	16	4.3
Asian	10	2.7
Native American	3	.8
Other	2	.5
Multiple	20	5.4
Ethnicity		
Not Hispanic	299	80.8
Hispanic	60	16.2
Not reported	11	3.0
Annual Household Income		
$0 - $24,999	37	10.0
$25,000 - $49,999	63	17.0
$50,000 - $74,999	69	18.6
$75,000 - $99,999	64	17.3
$100,000 - $149,999	61	16.5
$150,000 or more	46	12.4
Not reported	30	8.1
Comorbidities		
Agoraphobia	25	6.8
Alcohol use disorder	21	5.7
Attention-deficit/hyperactivity disorder	115	31.1
Autism spectrum disorder	35	9.5
Bipolar disorder	29	7.8
Body dysmorphic disorder	26	7.0
Borderline personality disorder	15	4.1
Eating disorder	54	14.6
Generalized anxiety disorder	194	52.4
Hoarding disorder	4	1.1
Major depressive disorder	186	50.3
Obsessive-compulsive disorder	75	20.3
Panic disorder	54	14.6
Post-traumatic stress disorder	45	12.2
Schizophrenia	7	1.9
Social anxiety disorder	107	28.9
Substance use disorder	15	4.1
Tic disorder	10	2.7
Other	1	11
None	40	10.8
Substance use (in the past year)		
Amphetamines	16	4.3
Anabolic steroids	0	100.0
Cannabis	130	35.1
Cocaine	26	7.0
Dissociative drugs	18	4.9
Hallucinogens	45	12.2
Inhalants	9	2.4
Opiates	23	6.2
Sedatives	15	4.1
Other	1	0.3
None	197	53.2
Medications		
Antidepressants	161	43.5
Antipsychotics	27	7.3
Anxiolytics	80	21.6
Medications prescribed for ADHD	69	18.6
Medications prescribed for BFRBs	19	5.1
Mood stabilizers	26	7.0
Sleep aids	35	9.5
Other	4	1.1
None	131	35.4

ADHD, attention-deficit/hyperactivity disorder; BFRB, body-focused repetitive behaviors.

**Figure 1:**
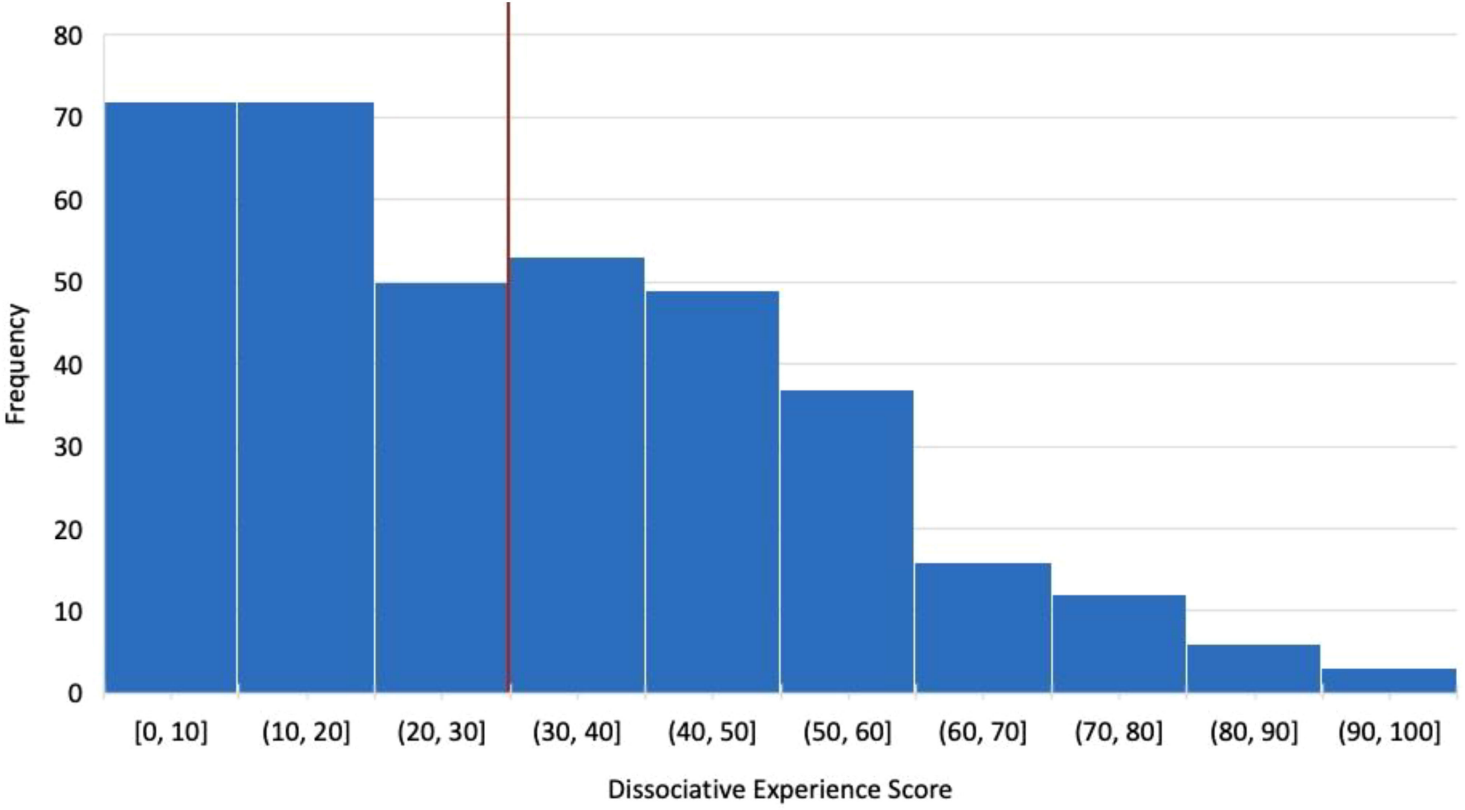
Dissociative experience score distribution.

Of the 370 participants, 99 had trichotillomania (26.8%), 176 had skin picking disorder (47.5%), and 95 had comorbid trichotillomania and skin picking disorder (25.7%). The mean DES score for trichotillomania, skin picking disorder, and comorbid trichotillomania plus skin picking disorder were 35.8, 28.1, and 32.7, respectively (**Table 2**). Notably, the full sample’s mean DES score was 31.3 (SD = 21.5), with 178 participants (48.1%) scoring above the 30 point cutoff for high dissociation (**Figure 1**).

**Table 2:** Descriptive Statistics of the Dissociative Experience Scale.

Sample	N	%	DES	
			M	SD
All	370	100.0	31.3	21.5
TTM	99	26.8	35.8	25.3
SPD	176	47.5	28.1	20.0
TTM and SPD	95	25.7	32.7	19.0

DES, Dissociative Experience Scale; TTM, trichotillomania; SPD, skin picking disorder; M, mean; SD, standard deviation.

### 3.2 Linear regression analysis predicting GBS-8 score

Linear regression was used to analyze whether dissociation (DES score) was associated with severity of trichotillomania and skin picking disorder using the GBS-8 total score. The results suggested that the predictive model was statistically significant, where DES score explained 11% of the variance of GBS-8 score (R^2^ = .110, F = 45.616, *p* <.001). DES score appeared to predict GBS-8 score, β = .068, t = 6.754, *p* <.001. Similar findings were reported when looking at the DES subscales and their association with the GBS-8: amnesia (R^2^ = .084, F = 33.662, *p* <.001; β = .052, t = 5.802, *p* <.001); depersonalization/derealization (R^2^ = .091, F = 36.645, *p* <.001; β = .053, t = 6.054, *p* <.001); absorption (R^2^ = .094, F = 38.127, *p* <.001; β = .059, t = 6.175, *p* <.001) (**Table 3**).

**Table 3:** Linear regression: DES score predicting GBS-8 score.

Variable	β	SE	95% CI		P
			LL	UL	
DES	.068	.010	.048	.088	<.001*
Amnesia	.052	.009	.035	.070	<.001*
Depersonalization/Derealization	.053	.009	.036	.071	<.001*
Absorption	.059	.010	.040	.078	<.001*

* indicates p < 0.05, n = 370.

DES, Dissociative Experience Scale; GBS-8= Body-Focused Repetitive Behaviors Scale-8; SE, standard error; CI, confidence interval; LL, lower limit; UL, upper limit.

### 3.3 Regression analysis predicting clinical outcomes

Further regression analysis suggested that DES scores were also associated with clinical outcomes related to compulsive behaviors. More specifically, DES score was associated with GBS-8 impairment score (R^2^ = .199, F = 91.502, *p* <.001; β = .057, t = 9.566, *p* <.001), but not GBS-8 severity. Several binary logistic regressions were performed to investigate whether DES score could predict factors related to suicidality. The odds ratios for each regression were: suicidal ideation (Exp(β) = 1.021, 95% CI [1.008, 1.342], *p* = .001), non-suicidal self-injury (Exp(β) = 1.025, 95% CI [1.014, 1.035], *p* <.001), and history of suicide attempt (Exp(β) = 1.033, 95% CI [1.021, 1.044], *p* <.001) (**Table 4**).

**Table 4:** Regression analyses: DES score predicting clinical outcomes.

Effect	N	β	SE	Wald	95% CI for β		Exp(β)	P
					LL	UL		
GBS-8 Severity^a^	370	.011	.006		.000	.022		.058
GBS-8 Impairment^a^	370	.057	.006		.046	.069		<.001*
Suicidal Ideation^b^	370	.021	.007	10.117	1.008	1.034	1.021	.001*
Non-Suicidal Self-Injury^b^	369	.024	.005	20.885	1.014	1.035	1.025	<.001*
Suicide Attempt^b^	368	.032	.006	32.105	1.021	1.044	1.033	<.001*

* indicates p < 0.05.

^a^Linear regression, ^b^Binary logistic regression.

DES, Dissociative Experience Scale; GBS-8= Body-Focused Repetitive Behaviors Scale-8; SE, standard error; CI, confidence interval; LL, lower limit; UL, upper limit.

## 4 Discussion

Consistent with previous literature, these findings suggest that individuals with trichotillomania (35.8 ± 25.3) and skin picking disorder (28.1 ± 20.0) report greater severity of dissociation (based on DES total scores) than a non-clinical adult population (11.57 ± 10.63) and a general psychiatric patient population (16.67 ± 16.41) (16). The DES scores for this study are more aligned with that of other dissociative disorders, which collectively have a mean DES of 41.22 ± 21.99 (16). These findings are consistent with many clinical observations of trichotillomania and skin picking disorder patients who often report being unable to recall how long or even if they have been pulling and picking. Interestingly, our sample also had higher mean DES scores than reported in two previous studies of trichotillomania and skin picking disorder (11, 12). This could be due to a unique feature of the participants in this study or the significantly larger sample size. These new findings may suggest that people with trichotillomania and skin picking disorder experience dissociation at a greater level than previously reported in the literature.

Additionally, this study explored the impact of dissociation on trichotillomania and skin picking disorder by examining several clinical variables and found that dissociation, as measured by DES score, was associated with greater impairment, higher likelihood of self-injury, and increased suicidal ideation and attempts. There was not a significant relationship between pulling and picking severity and DES scores, however. This finding might suggest that some people who experience dissociation may not recall the urges or time spent pulling and picking (which the GBS-8 uses to measure severity). While one theory is that dissociation results in worse pulling and picking, it is also plausible that hair pulling or skin picking creates a trance-like state for patients in which they find themselves dissociating.

It is important to be cautious when applying these study findings as they lack longitudinal data and are limited to associations that cannot determine causality. Additionally, this study relied on self-reported data; thus, findings may be impacted by participants over-reporting or under-reporting of diagnoses and symptoms. Future research should aim to elucidate the causal factor in this relationship to further characterize the association and determine whether treatment of dissociation is warranted. Studies of impulsive self-injurious behavior in borderline personality disorder patients and eating disorder patients have suggested that impulse control may be mediated, in part, by dissociative experiences (17). Therefore, understanding the complex interplay of impulsivity, dissociation, and pulling and picking may be a useful future direction.

A potential limitation of this study is the participants were not assessed for anxiety symptoms. Episodic anxiety in non-dissociative psychiatric disorders may artificially elevate DES scores. The experience of depersonalization and derealization has long been hypothesized to occur during states of anxiety (18). Trichotillomania and skin picking disorder often have a prominent component of anxiety (19), and the question arises whether the elevated DES scores merely reflect elevated anxiety. Finally, the inability to analyze certain clinical outcomes of interest such as presence of comorbidities and substance use, due to poor fit of their regression models. These would provide more insight into the impact that dissociation has on patients with compulsive behaviors beyond this study.

In summary, these preliminary data suggest that adults with trichotillomania and skin picking disorder experience dissociative symptoms, as reflected on the DES scale, to a significant degree in many cases. Further studies, possibly with other scales of dissociation, are needed to elucidate further the cognitive experiences of these people.

## Data Availability

The raw data supporting the conclusions of this article will be made available by the authors, without undue reservation.
